# Safety and efficacy of topiramate in neonates with hypoxic ischemic encephalopathy treated with hypothermia (NeoNATI)

**DOI:** 10.1186/1471-2431-12-144

**Published:** 2012-09-05

**Authors:** Luca Filippi, Patrizio Fiorini, Marta Daniotti, Serena Catarzi, Sara Savelli, Claudio Fonda, Laura Bartalena, Antonio Boldrini, Matteo Giampietri, Rosa Scaramuzzo, Paola Papoff, Francesca Del Balzo, Alberto Spalice, Giancarlo la Marca, Sabrina Malvagia, Maria Luisa Della Bona, Gianpaolo Donzelli, Francesca Tinelli, Giovanni Cioni, Tiziana Pisano, Melania Falchi, Renzo Guerrini

**Affiliations:** 1 Neonatal Intensive Care Unit, Medical Surgical Feto-Neonatal Department, “A. Meyer” University Children’s Hospital, Viale Pieraccini, 24, I-50139, Florence, Italy; 2 Department of Radiological Sciences, “A. Meyer” University Children's Hospital, Viale Pieraccini, 24, I-50139, Florence, Italy; 3 Neonatal Unit, University of Pisa, Via Roma, 67, I-56126, Pisa, Italy; 4 Istituto di Scienze della Vita, Scuola Superiore Sant’Anna, Piazza Martiri della Libertà, 33, I-56127, Pisa, Italy; 5 Pediatric Emergency and Intensive Care, Department of Pediatrics, La Sapienza University of Rome, Viale del Policlinico, 155, I-00186, Rome, Italy; 6 Division of Child Neurology, Department of Pediatrics, La Sapienza University of Rome, Viale del Policlinico, 155, I-00186, Rome, Italy; 7 Department of Pharmacology, University of Florence, Viale Pieraccini, 6, I-50139, Florence, Italy; 8 Laboratory for diseases of the nervous system and metabolism, “A. Meyer” University Children’s Hospital, Viale Pieraccini, 24, I-50139, Florence, Italy; 9 Department of Sciences for Woman and Child's Health, University of Florence, “A. Meyer” Children's Hospital, Viale Pieraccini, 24, I-50139, Florence, Italy; 10 Department of Developmental Neuroscience, Stella Maris Scientific Institute, Viale del Tirreno, 331, I-56128, Calambrone, Pisa, Italy; 11 Paediatric Neurology Unit and Laboratories, “A. Meyer” Children’s Hospital, University of Florence, Viale Pieraccini, 24, I-50139, Florence, Italy

**Keywords:** Neonatal hypoxic-ischemic encephalopathy, Therapeutic hypothermia, Topiramate

## Abstract

**Background:**

Despite progresses in neonatal care, the mortality and the incidence of neuro-motor disability after perinatal asphyxia have failed to show substantial improvements. In countries with a high level of perinatal care, the incidence of asphyxia responsible for moderate or severe encephalopathy is still 2–3 per 1000 term newborns. Recent trials have demonstrated that moderate hypothermia, started within 6 hours after birth and protracted for 72 hours, can significantly improve survival and reduce neurologic impairment in neonates with hypoxic-ischemic encephalopathy. It is not currently known whether neuroprotective drugs can further improve the beneficial effects of hypothermia. Topiramate has been proven to reduce brain injury in animal models of neonatal hypoxic ischemic encephalopathy. However, the association of mild hypothermia and topiramate treatment has never been studied in human newborns. The objective of this research project is to evaluate, through a multicenter randomized controlled trial, whether the efficacy of moderate hypothermia can be increased by concomitant topiramate treatment.

**Methods/Design:**

Term newborns (gestational age ≥ 36 weeks and birth weight ≥ 1800 g) with precocious metabolic, clinical and electroencephalographic (EEG) signs of hypoxic-ischemic encephalopathy will be randomized, according to their EEG pattern, to receive topiramate added to standard treatment with moderate hypothermia or standard treatment alone. Topiramate will be administered at 10 mg/kg once a day for the first 3 days of life. Topiramate concentrations will be measured on serial dried blood spots. 64 participants will be recruited in the study. To evaluate the safety of topiramate administration, cardiac and respiratory parameters will be continuously monitored. Blood samplings will be performed to check renal, liver and metabolic balance. To evaluate the efficacy of topiramate, the neurologic outcome of enrolled newborns will be evaluated by serial neurologic and neuroradiologic examinations. Visual function will be evaluated by means of behavioural standardized tests.

**Discussion:**

This pilot study will explore the possible therapeutic role of topiramate in combination with moderate hypothermia. Any favourable results of this research might open new perspectives about the reduction of cerebral damage in asphyxiated newborns.

**Trial registration:**

Current Controlled Trials ISRCTN62175998; ClinicalTrials.gov Identifier NCT01241019; EudraCT Number 2010-018627-25

## Background

### Neonatal hypoxic-ischemic encephalopathy: disease incidence and pathogenesis

In countries with a high level of perinatal care, the incidence of asphyxia responsible for moderate or severe encephalopathy is 2–3 per 1000 term infants [1,2]. The ensuing encephalopathy may present with need for resuscitation at birth, neurological depression, seizures or electroencephalographic (EEG) abnormalities. Hypoxic ischemic encephalopathy (HIE) is still the leading cause of perinatal mortality and severe neurological impairment. Mortality rate is 10% for moderate and 60% for severe HIE. About 30% of survivors with moderate and 100% with severe HIE exhibit permanent neurological disability [3,4].

Encephalopathy results from the combination of reduced cerebral oxygenation (hypoxemia) and/or reduced perfusion (ischemia). Cerebral damage develops in two distinct phases. During the acute phase neuronal death occurs by necrosis of neuronal cells, a result of a rapid depletion of brain energy. However, after a few hours, cerebral energy metabolism recovers, but a cascading series of biochemical events triggers neuronal apoptosis and leads to severe brain damage. These events include an increased release of excitatory neurotransmitters, especially glutamate [5]. Glutamate acts on three classes of receptors that control the passage of ion channels through the neural cell membrane: alpha-amino-3-hydroxy-5-methyl-4-isoxazolepropionate (AMPA), kainate and *N*-methyl-D-aspartate (NMDA) receptors. Neurons (and glia) contain high concentrations of glutamate, which is usually released in small amounts and for short periods of time (milliseconds) [6]. Exceedingly high glutamate concentrations can over-excite nervous cells and lead to their death (excito-toxicity) [7]. This excito-toxicity contributes to neuronal damage in various neurodegenerative disorders.

Glutamate activates AMPA receptors, depolarizes the cell and promotes the removal of the voltage-dependent block operated by Mg^++^[8,9] on NMDA receptors: this promotes the entry of Ca^++^ through this channel [10]. Calcium entry stimulates in turn a series of processes that lead to necrosis and apoptosis. These processes include the Ca^++^ overload in the mitochondria, responsible for the production of free radicals [6,11-13]; the activation of caspases and the release of factors inducing apoptosis [14-17]; the activation of a neuronal nitric oxide synthase (nNOS) that triggers the synthesis of nitric oxide (NO) and the formation of toxic peroxynitrite and nitrosylated-GAPDH; the stimulation of p38 mitogen-activated protein kinase (p38 MAPK), which activates the transcription of factors that enter the nucleus and promote neuronal damage and apoptosis [18-22].

Between the acute initial damage and the ensuing damage mediated by the release of toxic substances, there is therefore a time window that could offer the possibility of a combined neuroprotective treatment.

### Hypothermia as a neuroprotective treatment in animals

Currently the most effective neuroprotective intervention for asphyxiated infants is treatment with moderate hypothermia, a reduction in body temperature of 3-4°C. Experimental studies performed in adult and newborn animals have shown that reducing brain temperature by 2-4°C after ischemia protects the brain from neuronal damage and cell death and improves neurological [23-26] and behavioral outcome, as well as histological changes [27-29]. Several mechanisms may explain these benefits: hypothermia reduces the accumulation of extracellular glutamate, enhancing post-ischemic glutamate re-uptake [30], the synthesis of free radicals and nitric oxide [15], preserves the content of high-caloric cerebral phosphates, reduces cerebral alkalosis and lactate levels [16,31]. Hypothermia may also suppress the activation of microglia [32-34]. These actions can preserve brain energy metabolism, reduce cytotoxic cerebral edema and inhibit neuronal death. Thanks to animal experiments, the optimal temperature for brain neuroprotection, leading to reduce biochemical and histological markers of neuronal damage, has been observed to be between 32 and 34°C [13,35-37]. Most of these studies indicate that hypothermic treatment should be immediate or precocious (within 6 hours from the insult), while neuroprotection diminishes or disappears if the cooling is delayed beyond 6 hours [27,38-42]. For this reason, hypothermic treatment following asphyxia should be initiated as soon as possible. The duration of treatment with hypothermia varied in such experimental studies. Significant benefits were obtained when moderate hypothermia was maintained for at least 12–24 hours, but it has been suggested that more prolonged treatment, up to 72 hours [42], may be necessary if the interval before induction of hypothermia is prolonged [43]. In sheep fetuses a rebound of epileptic activity has been reported after rapid heating [43]. Finally, hypothermia appears to be more neuroprotective in animal exposed to moderate, rather than severe asphyxia [40,44].

In conclusion, there is a strong experimental evidence that moderate hypothermia in asphyxiated animals produces behavioral and histological benefits both in the short-term [40,45], and in the long-term [27,46-49].

### Hypothermia as a neuroprotective treatment in human neonates

In human neonates hypothermia has been described for the first time as a treatment for resuscitation in the '60s, before modern techniques of resuscitation were introduced [50]. In such occasions, hypothermia was used only for short periods of time after failed resuscitation, but details regarding side-effects or clinical long-term outcome of these infants were not provided [51-55]. Since the late '90s, on the basis of the above mentioned animal studies, pilot studies with selective brain hypothermia associated with generalized mild hypothermia or generalized moderate hypothermia between 35.5 and 33.0°C for 48–72 hours, have been performed in infants with HIE demonstrating the feasibility of this treatment in term infants [56-62]. In the early 2000, the first cases reports showed better neurodevelopmental outcomes in cooled asphyxiated infants: whole-body hypothermia was observed to reduce the damage of thalami, basal ganglia, and white matter, while selective brain hypothermia appeared to be more effective in protecting the cortex [63-65].

So far, seven randomized controlled trials regarding the efficacy of therapeutic hypothermia for the treatment of neonatal encephalopathy have been published [66-72]. Two such trials adopted the selective cerebral hypothermia [66,72] and five the total-body hypothermia [67-71]. All of these trials, powered to detect a difference in the primary composite outcome of death and/or disability, demonstrated the benefits of hypothermia. A meta-analysis of the first trials showed that moderate hypothermia reduced death or disability at 18 months with a risk ratio of 0.81 (95% CI, 0.71-0.93) and a number needed to treat (NNT) of 9 [73]. The first data from follow-up of newborns enrolled in the CoolCap Trial and NICHD Trial showed that neurodevelopmental outcomes calculated at 18–22 months of life predicted the functional outcomes into childhood [74,75].

Overall, if the small study of Eicher is excluded [76], major trials suggest that cooling is a safe procedure in newborns. Recent reviews concluded that bradycardia and thrombocytopenia were the only clinical, though benign, adverse effects that were more common with hypothermia [77-79]. Therapeutic hypothermia has recently been included in the Guidelines of International Consensus on Cardiopulmonary Resuscitation and Emergency Cardiovascular Care Science and in the recommendations of the American Heart Association [80,81].

Neuroprotective drugs may enhance the efficacy of hypothermia for the treatment of neonatal HIE [82]. However, there are no studies that have explored the combined treatment with hypothermia and topiramate in asphyxiated newborns.

### Topiramate

Topiramate (TPM) is an anticonvulsant agent widely used in adults and children, characterized by good absorption, high bioavailability, and good tolerability [83]. TPM has multiple mechanisms of action, including glutamate-receptors inhibition [83,84], which implies a potential as neuroprotective agent. TPM did indeed demonstrate neuroprotective properties against hypoxic ischemic brain damage, both *in vitro* and in animal models, and was recently proposed as an innovative neuroprotective therapy for ischemic stroke [85-92] and neonatal hypoxic-ischemic cerebral injury [93]. In neuronal cultures, cell damage induced by oxygen-glucose deprivation [91] or excitotoxic glutamate or kainate concentrations [94], was consistently attenuated by TPM. In animal models of transient global cerebral ischemia intravenous, intraperitoneal, or oral TPM reduced the severity of cerebral damage either alone [86-89] or with hypothermia [90] in a dose-dependent manner, with neuroprotective doses ranging from 5–200 mg/kg, usually in single administration [87-91]. TPM was also demonstrated to exert neuroprotective effects against periventricular leukomalacia [92].

The neuroprotective mechanisms of TPM appear to be related not only to AMPA and kainate receptors inhibition [92,94-97], but also to blockade of Na^+^ channels [98], high voltage-activated calcium currents [85], carbonic anhydrase isoenzymes [99], and mitochondrial permeability transition pore (MPTP) [100].

To date, no clinical study has been published to prove an additive or synergistic action of TPM combined with hypothermia in newborns with HIE.

We previously reported that TPM pharmacokinetic properties at the dose of 5 mg/kg appear to be modified by concomitant hypothermia [101]. Likewise observed with other poorly metabolized drugs [102], hypothermia reduces TPM clearance and slows absorption and elimination processes [103].

Although long-term effects on cognitive functions of TPM administration in early life remain to be assessed, short-term safety is reassuring enough to support its evaluation in clinical trials that explore its possible additive neuroprotective action [104]. The gap between effective and neurotoxic doses is greater for TPM than for other antiepileptic drugs [103], and short-course therapy appears to have few neurotoxic effects. Regarding TPM long-term effects, in asphyxiated animal models treated with TPM, no cognitive deficit was demonstrated [91], and in epileptic neonate rodents, TPM was safer than phenobarbital or benzodiazepines [103,105]. Neuronal death occurred at doses of 50 mg/kg, which are considerably higher than doses used in common therapeutic schedules.

### Hypothesis

In conclusion, several *in vitro* and *in vivo* experimental studies have demonstrated that both, hypothermia and TPM, are able to reduce post-ischemic neuronal damage. So far no study has investigated whether the combined action of these procedures may be additive to their individual neuroprotective potential. We hypothesize that the combination treatment with moderate whole-body hypothermia associated with TPM administration is safe and enhances the neuroprotective properties of hypothermia for the treatment of neonatal HIE.

### Objectives

#### Major objectives: safety and efficacy of TPM associated with moderate whole-body hypothermia

The first purpose of this study is to confirm the safety of TPM administration in asphyxiated newborns. For this purpose, cardiac and respiratory parameters (heart frequency, blood pressure, oxygen saturation, respiratory support), will be continuously monitored. Blood samplings will be performed to check renal, liver and metabolic balance.

TPM is considered to be safe and generally well tolerated in children. The safety profile of TPM might be different in neonates with HIE treated with hypothermia than in adults receiving chronic therapy for epilepsy. TPM can cause metabolic acidosis, especially in pediatric patients, because of carbonic anhydrase inhibition at proximal renal tubule with consequent renal loss of bicarbonate [106,107]. In as many as 48% of adults and 67% of children with epilepsy treated with TPM, a variable degree of metabolic acidosis develops. However, in most reported cases, bicarbonate levels did not decrease to be clinically significant. Only 11% of patients had serum bicarbonate levels <17 mEq/L [106,108] and symptomatic cases were successfully treated with sodium bicarbonate supplementation [106]. Although this observation is reassuring, metabolic acidosis is usually severe in neonates with HIE, who have metabolic acidosis because of both asphyxia and renal impairment. In infants, metabolic acidosis usually occurs after 8 to 26 days of TPM treatment, with dosages as high as 8.2 to 26 mg/kg/day [109]. Similar to other carbonic acid anhydrase inhibitors, long-course TPM therapy is associated with increased risk of nephrolithiasis in adults [106,107]. Angle closure glaucoma and acute myopia are additional, though potentially reversible, adverse events related to TPM treatment in the pediatric age [106,107,110]. In a safety study, we found no signs of metabolic acidosis in newborns co-treated with moderate hypothermia and TPM; abdomen ultrasound scanning did not show kidney stones and ophthalmological evaluation was normal in all treated newborns [104].

The second objective of this study is to evaluate the efficacy of TPM add-on therapy, evaluating the clinical outcome of enrolled newborns. The neurological and neuroradiologic outcome of newborns treated with hypothermia plus TPM will be compared to the outcome of control group that received hypothermia alone.

## Methods/Design

### Study population-setting

Term newborns delivered at gestational age higher than 36 weeks and with birth weight higher than 1800 g admitted for HIE to the Neonatal Intensive Care Units (NICUs) of A. Meyer University Children’s Hospital, Florence, of the Santa Chiara University Hospital of Pisa and of the La Sapienza University of Rome.

### Study design

Multicenter interventional pilot randomized controlled trial to compare the safety and efficacy of TPM add-on therapy associated to the conventional approach (treatment with whole-body moderate hypothermia).

#### Hypothermia

All newborns who will satisfy admission criteria will be treated with moderate (33.5°C) hypothermia for 72 hours. Outborn patients will be initially cooled to 35°C at the birth hospital, avoiding heating and using ice packs during the transfer to NICU. In all the centers a cooling blanket with an esophageal probe will be used to induce hypothermia; the esophageal temperature will be lowered by the blanket’s servomechanism. Rectal temperature will be monitored by a rectal probe connected to cardiomonitor. After 72 hours of hypothermia, all newborns will be gradually re-warmed up to 36.5-37°C over the following 6–12 hours (0.5°C/h). Vital parameters will be continuously monitored under hypothermia.

Half of these newborns will be subjected to additional treatment with oral topiramate.

#### Topiramate

TPM (Topamax, Janssen-Cilag, Cologno Monzese, Milan, Italy) will be administered by orogastric tube as enteric-coated granules mixed with water at the beginning of hypothermia for the first three days of life, for a total amount of 3 doses per patient. Newborns will receive 10 mg/kg once a day, a dosage usually used in infants [111-113] and neonates [114-117]. TPM plasma concentrations will be measured as previously reported [118].

#### Monitoring

The following respiratory and hemodynamic parameters will be registered before starting hypothermia and then at hours 0, 6, 12, 18, 24, 30, 36, 42, 48, 54, 60, 66, 72 of hypothermia, and after the re-warming process: respiratory rate, oxygen saturation (SaO_2_), fractional inspired oxygen (FiO_2_), systolic, diastolic and mean arterial pressures, heart rate. The following blood tests will be performed in all newborns at hypothermia hours 0, 24, 48, 72 and after re-warming process: blood gas analysis (corrected for body temperature), serum electrolytes, liver and renal function tests, creatine-kinase, creatine-kinase MB isoenzyme, lactate dehydrogenase, troponin IC, complete cell blood count, C-reactive protein, procalcitonin and coagulation tests.

#### Clinical course and concomitant treatments

A central venous line will be placed in all patients. Fluid intake will be started at 60–70 mL/kg and increased of 10–20 mL/kg each day, basing on changes in body weight and serum electrolytes levels. Minimal enteral feeding will be allowed with human milk from the first day of life. In case of respiratory failure newborns will be put on patient triggered ventilation. Neonates who will develop seizures will be treated with phenobarbital, (loading dose 20 mg/kg, followed by 1.5-2.5 mg/kg every 12 hours). In case of resistance to phenobarbital, midazolam will be used with a dose-initial bolus of 0.15 mg/kg, followed by a continuous infusion (1 μg/kg/min) increased by 0.5-1 μg/kg/min every 2 minutes until a favourable response and up to a maximum dose of 18 μg/kg/min [117]. In case of hypotension, defined as a mean arterial blood pressure <40 mmHg, single or multiple normal saline boluses will be administered (10–20 mL/kg), and in case of refractoriness, dopamine, dobutamine, norepinephrine or terlipressin [119] will be progressively added. Newborns will receive analgesia with Fentanyl at a dose of 1 μg/kg/h, since hypothermia causes agitation and reducing the threshold of pain.

### Neurological follow-up

The duration of follow-up necessary for possible neuro-motor disabilities and cognitive loss will be 24 months.

Every newborn will be evaluated beyond the neonatal period, at 1, 3, 6, 12, 18 and 24 months of life. Standard EEG will be evaluated within one week and will be repeated if abnormal. Within the first week and three months of age, newborns will be studied with Neonatal Hammersmith neurological examination and General Movement (GMs) assessment [120]. The Hammersmith Infant Neurological Examination [121,122] will be performed between 3 and 6 months of life. The mental and motor development will be evaluated with the Bayley Scales of Infant and Toddler Development 3^rd^ edition [123] measure at 12, 18 and 24 months. Bayley scales will be used to measure the major areas of development: cognitive, language, motor, social-emotional and adaptive functioning.

Visual function will be assessed in the first ten days of life by means of a recently published battery of behavioural tests designed to assess various aspects of visual function [124,125], which includes items that assess ocular movements (spontaneous behaviour and in response to a target), the ability to fix and follow a black/white target (horizontally, vertically, and in an arc), the reaction to a colored target, the ability to discriminate between black and white stripes of increasing spatial frequency, and the ability to keep attention on a target that is moved slowly away from the infant. Visual function will be evaluated again at 4 ½, 6 and 12 months with particular regards to binocular visual acuity, measured by means of standardized instruments based on preferential force choice (Teller acuity cards), stereopsis and ocular motricity.

Acoustic function will be monitored until 24 months.

Neurologists responsible of clinical follow-up will be blindfolded about which newborns have been treated with TPM in addition to hypothermia.

#### Neuroradiologic follow-up

Standard cerebral magnetic resonance imaging (MRI), diffusion tensor imaging (DTI) and spectroscopy will be performed at the end of the hypothermic treatment within the first week, at three months and at 18 months.

Neuroradiologists responsible of follow-up will be blindfolded about which newborns have been treated with TPM in addition to hypothermia.

### Inclusion criteria

The treatment with therapeutic hypothermia will be reserved to newborns with gestational age ≥ 36 weeks and birth weight ≥ 1,800 g who will fulfill the following criteria:

1. Metabolic criteria: Apgar score ≤5 at 10 min, or persisting need for resuscitation, including endotracheal intubation or mask ventilation for more than 10 min after birth, or acidosis (pH ≤7.0 and/or base deficit ≥ −16 mmol/L in umbilical cord blood or arterial, venous, or capillary blood) within 60 min from birth;

2. Neurological criteria (modified from Sarnat and Sarnat [126]): moderate to severe encephalopathy consisting of altered state of consciousness (irritability, lethargy, stupor, or coma) and ≥ 1 of the following signs: hypotonia, or abnormal reflexes including oculomotor or pupil abnormalities, or absent or weak suctioning, or clinical seizures;

3. aEEG criteria: “moderately abnormal” indicates a background tracing with upper margin >10 μV and lower margin ≤5 μV; “severely abnormal” pattern refers to a tracing with upper margin <10 μV and lower margin <5 μV; often this is accompanied by bursts of high voltage activity (“burst suppression”). Seizures are identified as periods of sudden increase in voltage, accompanied by narrowing of the band of aEEG activity followed by a brief period of suppression or by a build up of rhythmic activity of increasing amplitude and decreasing frequency [127].

### Selection criteria for study subjects

At least one of the parents of the newborns meeting the inclusion criteria will be approached by the study investigator/nurse and informed of the study. A signed parental informed consent must be obtained.

### Exclusion criteria

1. Newborns with gestational age less than 36 weeks, with birth weights less than 1800 g, or admitted at the NICU after 6 hours of life.

2. Newborns with major congenital abnormalities or other syndromes that include brain malformations, congenital viral infections or evidence encephalopathy other than HIE.

3. Informed Consent refused.

### Allocation of participants to the trial groups (Randomization)

EEG is useful in identifying suitable newborns for hypothermia due to its capability to distinguish newborns with Sarnat grade 2 from those with Sarnat 1. Several studies have demonstrated that aEEG is easier to be interpreted than standard EEG, but equally accurate and reproducible. It correlates well with neurodevelopmental outcome of full-term infants with HIE, and it is considered the best single predictor of neurologic outcome. In some studies aEEG was more specific and exhibited a higher positive predictive value when compared with the neurologic examination performed in the first hours after delivery [128-131].

In all three recruiting hospitals, randomization will be done stratifying eligible newborns according to aEEG into moderate or severe HIE. Newborns will be assigned to the group of moderate or severe aEEG to ensure maximal clinical homogeneity between the groups.

Newborns will be randomized in blocks of eight for each group, alternating newborns between TPM added to standard hypothermic treatment and standard treatment alone.

### Experimental plan and data analysis

Based on the medical literature, the mean incidence of the combined frequency of mortality and severe neurodevelopmental disability in survivors at 18 months of age is approximately 50%. In fact this percentage is 55% in the cool Cap study [66], 44% in the NICHD study [68], 45% in the TOBY trial [69], 51.4% in the ICE trial [70], and 50.9% in the neo.nEURO.network study [71]. We hypothesize that treatment with TPM may reduce the incidence of the combined frequency of mortality and severe neurodevelopmental disability in survivors at 18 months at 15%. In order to compare the incidence of this primary outcome between newborns receiving TPM plus hypothermia (treated) and those only receiving hypothermia (control group), the estimated sample size was calculated, considering normal distribution, an alpha error of 0.05 and a power of 80 percent. The sample size for each group is 32 participants.

The three units have an overall admission rate of approximately 10 term newborns with HIE per year. Due to the high hypothetical advantages of this treatment, we estimate 90-100% rate of consent and we predict we would recruit the 64 newborns over around 24 months period with a realistic safety margin.

Mortality, the incidence of severe neuromotor disability, cortical vision, sensorineural hearing loss, developmental delay, epilepsy will be evaluated at 18 months of life.

To evaluate TPM safety, cardiac and respiratory parameters (heart frequency, blood pressure, oxygen saturation, respiratory support), will be continuously monitored. Blood samplings will be performed to check renal, liver and metabolic balance. Kruskal-Wallis test will be used to assess possible differences between newborns treated or not with TPM. The safety will be also evaluated by means of relative risk (RR) [132]. RR will be calculated as the ratio between the probabilities of side effects in the TPM group with respect to the control group. Values of RR lower than 1, will be associated to the efficacy of the treatment.

Finally, we will evaluate the different distribution of any brain lesions on MRI brain highlighted.The RR is calculated for each item studied. An RR <1 indicates an improvement of neurological, neurodevelopmental and neuroradiologic hypothermia due to the association-topiramate.

### Primary outcome

The neurological and neuroimaging outcome of these two groups of infants will be compared to determine whether adjunctive treatment with TPM improves the neuroprotective effect of hypothermic treatment.

The primary outcome will be the combined frequency of mortality and severe neurodevelopmental disability in survivors at 24 months of age. Severe disability is defined as Bayley III [123] cognitive development index 3 SDs below mean or any one of the components of severe sensorimotor disability (e.g. inability to walk, sit, feed using hands, communicate (Bayley III language development index 3 SDs below mean), hear (80 dB sensory neural hearing loss) or see.

### Secondary outcomes

The following secondary outcomes will also be assessed at 24 months of life:

•Multiorgan dysfunction (adverse events in three or more organ systems),

•Bilateral sensorineural hearing loss more than 40 dB

•Epilepsy (recurrent seizures beyond the neonatal, period requiring anticonvulsant treatment)

•Developmental delay

•Multiple Disabilities (epilepsy, cortical visual impairment, sensorineural hearing loss, developmental delay)

Finally, the neuroradiologic outcome will be assessed by comparing the imaging obtained by standard cerebral MRI, DTI and spectroscopy performed within the first 7 days, at 3 months and 18 months of age. Brain injuries will be classified as isolated lesions of the white matter (WM), of basal ganglia and thalami (BGT), with or without involvement of the posterior limb of the internal capsule (PLIC), of cortex (COR), or various combinations of such lesions [65].

### Ethical approval

A three-centre phase II pilot study entitled “Safety and Efficacy of Topiramate in Neonates With Hypoxic Ischemic Encephalopathy Treated With Hypothermia (NeoNATI)” has been approved by the Ethics Committees of A. Meyer University Children’s Hospital, Florence, of University of Pisa and of La Sapienza University, Rome (prot. 276/2010).

Informed consent will be obtained from at least one parent prior to study entry. Parents will be given full verbal and written information regarding the objective and procedures of the study and the possible risks involved.

TPM is considered safe for use in children. Nevertheless, a careful watch will be kept on all study participants with regard to side effects of drug administration. Parents of participants will be made aware of possible side effects. Infants will be monitored in the Neonatal Intensive Care Unit throughout the study period and their clinical condition will be evaluated daily as part of medical rounds. A letter informing the participant and the family doctor as to which study arm the participant had been randomized to will be sent following completion of the study.

In the presence of adverse events, a reduction of dosage will be taken into account.

For ethical and scientific reasons, an interim analysis after the enrolment of half of the newborns, is planned.

### Exit criteria

Patients will be withdrawn from the study if parents make specific request for the treatment to be discontinued before 72 hours, in case of severe bleeding, thrombosis, or pulmonary hypertension difficult to treat, arrhythmia, clinical electroencephalographic or neuroimaging evidence of irreversible severe brain damage.

### Measurement of outcomes

#### Primary endpoint

To evaluate if the TPM administration reduces the cerebral damage of newborns with HIE and treated with therapeutic hypothermia, neurological and neuroradiologic follow-up are planned at different stages.

#### Secondary endpoint

a. To evaluate the safety of TPM treatment, cardiac and respiratory parameters (heart frequency, blood pressure, oxygen saturation, respiratory support) will be continuously monitored; blood samplings will be performed to check renal, liver and metabolic balance

b. To evaluate if TPM improves the functional and structural outcome, visual function is planned at 40 weeks gestational age, 4 ½, 12, 18 and 24 months corrected age.

#### Confidentiality

The participants’ data collected during this trial will be kept confidential. Study staff will have access to the data as well as the participants’ medical records as they pertain to this study. Published results will not contain any information that would identify individual participants.

## Discussion

The objective of this research is highly ambitious: to find a treatment procedure that is simple, inexpensive, well tolerated and with few adverse effects, able to reduce post-ischemic neuronal damage. Neuroprotection is a major health care priority, given the enormous burden of human suffering and financial cost caused by perinatal brain damage. Hypothermic treatment is recognized as a neuroprotective treatment after a profound neonatal insult. However, it is clear from previously published clinical trials and animal studies that hypothermia alone does not provide complete protection. Neuroprotective drugs added during or after hypothermia might improve neurologic protection, by extending the therapeutic window or providing long-lasting additive or synergistic action [133]. This combination treatment could open new perspectives for the management of asphyxiated infants. To date, no clinical study has evaluated the efficacy of the combined treatment hypothermia and TPM, an antiglutaminergic drug mainly used for the treatment of epilepsy. On the other hand, it is important to consider that drugs administered during the neonatal period may be dangerous to the immature brain, and hypothermia can modify their pharmacokinetics. In fact, excretion of many drugs and their metabolites can be modified by hypothermia; failure of liver and kidney clearance due to hypoxic-ischemic injury could exacerbate any toxicity. In newborns with HIE, TPM pharmacokinetic properties appear to be modified by concomitant hypothermia, but the dosage of 5 mg/kg appears safe. In this study the dosage will be increased to 10 mg/kg, likewise in other studies. For this reason one of the main objectives of this study is to confirm the safety of TPM added to hypothermia.

## Abbreviations

HIE: Hypoxic ischemic encephalopathy; EEG: Electroencephalography; AMPA: Alpha-amino-3-hydroxy-5-methyl-4-isoxazolepropionate; NMDA: *N*-methyl-D-aspartate; TPM: Topiramate; MRI: Magnetic Resonance Imaging; DTI: Diffusion tensor imaging.

## Competing interests

The author(s) declare that they have no competing interests.

## Authors’ contributions

LF conceived the study, he is Chief Investigator, participated in the design and helped to draft the manuscript. MD, SC, helped to draft the manuscript. MD, SC, LB, AB, MG, RS, PP, participated in the study design and are responsible for data collection. TP, MF, AS, FDB participated in the study design and are responsible for the neurological follow-up. SS, CF participated in the study design and are responsible for the radiological follow-up. FT participated in the study design and is responsible for the visual function evaluations. GLM, SM, MDB participated in the design of the study and are responsible for the determination of TPM concentration on dried blood spots. PF, GC, GD, RG participated in the design of the study and reviewed the protocol. All authors contributed to the development of the protocol, and read and approved the final manuscript.

## Pre-publication history

The pre-publication history for this paper can be accessed here:

http://www.biomedcentral.com/1471-2431/12/144/prepub
